# Enhancing Serious Game Design: Expert-Reviewed, Stakeholder-Centered Framework

**DOI:** 10.2196/48099

**Published:** 2024-05-31

**Authors:** Lance Bunt, Japie Greeff, Estelle Taylor

**Affiliations:** 1 Unit for Data Science and Computing North-West University Vanderbijlpark South Africa; 2 Optentia North-West University Vanderbijlpark South Africa

**Keywords:** serious games, stakeholder theory, enterprise architecture, serious game development, design framework

## Abstract

**Background:**

Traditional serious game design methods often overlook stakeholder needs. This study integrates stakeholder theory and enterprise architecture (EA), along with the Architecture Development Method, to propose a novel framework for serious game design. Crafted to aid practitioners, researchers, and specialists in leveraging resources more effectively, the framework is validated through a design science research methodology. Expert reviews have further refined its features, making it a robust tool for enhancing serious game design and implementation.

**Objective:**

This paper introduces a framework for designing serious games, covering stakeholder analysis, requirements gathering, and design implementation planning. It highlights the importance of expert review in validating and refining the framework, ensuring its effectiveness and reliability for use in serious game design. Through critical assessment by experts, the framework is optimized for practical application by practitioners, researchers, and specialists in the field, ensuring its utility in enhancing serious game development. The next step will be to validate the framework empirically by applying it to a serious game development project.

**Methods:**

We developed and validated a conceptual framework for serious game design by synthesizing stakeholder theory and EA through literature review, concept mapping, and theory development by way of a design science research approach. The framework is iteratively refined and validated via expert review, drawing on insights from professionals experienced in serious games, stakeholder theory, and EA. This method ensures the framework’s practical relevance and effectiveness in addressing real-world design challenges.

**Results:**

An expert review by 29 serious game practitioners validated the framework’s success in stakeholder management, confirming its stakeholder-centered effectiveness. Although the experts praised its structured approach, they suggested clearer guidance for game design elements. In addition, the experts, while acknowledging the framework’s complexity, saw its depth as valuable for efficient management. The consensus calls for a refined balance between detailed functionality and user-friendly design, with the framework’s impact on stakeholder capabilities revealing a spectrum of professional needs.

**Conclusions:**

This paper presents a framework for creating effective and organizationally aligned serious games. Evaluated across execution, practical, and EA levels, it is logical but varies in ease of understanding, with experts calling for more accessibility at the EA level. It enhances stakeholder efficiency and management but is criticized for rigidity and a need for flexibility. Recommendations include streamlining the framework, enhancing clarity, reducing administrative tasks, and incorporating clear guidelines on technology use, motivational elements, and operational tools. This aims to help stakeholders produce more targeted and adaptable game designs. The next iteration will be developed after application to a project and team feedback.

## Introduction

### Background

This paper articulates a stakeholder-centered framework for serious game design in various stages before, during, and after various methods have been applied to develop it. The research presents the framework—informed by stakeholder theory and enterprise architecture (EA)—as it evolved through various stages of development. It underscores the need for a structured approach that focuses on stakeholders throughout the design process to enhance the success of serious game production efforts. This research reflects 3 cycles of the design science research (DSR) paradigm, aiming to balance domain-specific needs with generalizable solutions. The framework emphasizes alignment with stakeholder needs and effective communication among different groups, recognizing the complexity of serious games and the importance of their relevance to users.

This work also presents findings from an expert review questionnaire that used a qualitative methodology to assess the framework’s effectiveness. The review gathers feedback from practitioners and specialists in the field, guiding enhancements to the framework’s clarity, structure, and usability. The paper concludes with insights into the framework’s current state of development and recommendations for its refinement, emphasizing the need to simplify its complexity and communicate its components more effectively for real-world application.

### Framework Requirements

Several key requirements for the initial framework emerged from a comprehensive integrative literature review. A conceptual framework is indispensable for comprehending the complex phenomena explored because it provides a structured and systematic method for organizing, analyzing, and interpreting data. Several essential components of a conceptual framework contribute to its efficacy, including clarity, relevance, coherence, simplicity, testability, and generalizability. As such, the following characteristics of the framework that has been developed serve as guiding principles. First, a conceptual framework should be clear and straightforward for its intended audience to comprehend. It should define its key concepts, variables, and relationships as well as provide a thorough overview of the subject under investigation. Second, the framework should be pertinent to the research problem or question being addressed. It must thus be tailored to the context and objective of the study. Third, the framework must have logical coherence, a clear structure, and internal consistency. The framework’s concepts, assumptions, and relationships should be logically connected and consistent with one another. Fourth, a good conceptual framework should be as simple as possible while capturing the essential characteristics of the studied phenomenon. Fifth, the framework must be susceptible to empirical testing, with testable hypotheses and predictions that can be evaluated through observation and data analysis. Sixth and last, such a framework should be applicable to other settings or situations and be generalizable beyond the specific context of the study. It should therefore serve as a foundation for the development of broader theoretical insights and generalizations about the subject of study.

Our framework prioritizes stakeholder engagement and management within serious game design, addressing a gap often overlooked in conventional design literature. While incorporating established design elements—such as learning objectives [1-4], game mechanics [5-9], narrative [10,11], user interface and experience [12], and evaluation [13,14]—the framework’s novelty lies in its stakeholder-centered approach. It is tailored to align with educational or training standards, drive engagement, and provide meaningful feedback. However, the primary focus is not solely on game design; instead, the framework is rooted in stakeholder theory and EA, which have been the fulcrum of our extensive literature review. By doing so, we address the intricacies of organizational and stakeholder dynamics, ensuring that serious games are developed within a context that appreciates the diverse roles and impacts of various stakeholders.

### Products of the Integrative Literature Review

#### Overview

The integrative literature review presents a taxonomy of serious games; the phases of serious game production; the stakeholders involved in serious game production; stakeholder identification, analysis, and management procedures; and The Open Group Architecture Framework (TOGAF) Architecture Development Method (ADM). These concepts are briefly outlined in the following subsections because they inform the construction of the conceptual serious game framework.

#### What? Classifying Serious Games

Serious games are edifying artifacts, tools, and games created by development teams that use ludic activity for a specific purpose, format, genre, interaction style, and application area. Serious game taxonomies classify games by *purpose*. This classification can help identify the functions of a serious game and guide the selection and development of educational and training games. The following are some serious game categories:

- Simulation games simulate real-world situations to give learners practical experience and practice in complex or high-risk situations (eg, flight simulators for pilot training and medical simulators for surgery [15]).
- Educational games teach specific knowledge or skills, such as language, math, or history. Game mechanics such as rewards and feedback encourage learning and participation [16].
- Training games teach practical abilities such as customer service, leadership, and teamwork. To track progress and facilitate learning, they may include simulations or role-playing scenarios as well as feedback and assessment [17].
- Health games promote healthy behaviors such as exercise, healthy eating, and disease management. Game mechanics such as rewards and challenges may encourage behavior modification and participation [18].
- Persuasive games aim to influence players to adopt certain behaviors, such as environmental conservation, social justice, or political activism. Story elements often emotionally engage and motivate players [19].

By classifying serious games by their intended function, a functional taxonomy can help find the best games for learning or training needs and guide their development. These serious game classifications are relevant to this research and accepted under the serious game banner.

Serious games, gamification, and game-based learning differ greatly. These terms all refer to using games or game elements in learning or training, but serious games are games with a specific goal. They usually teach players a skill [20]. Serious games differ from entertainment-focused commercial games. Gamification, by contrast, uses game elements such as points, badges, and leader boards to motivate and engage nongame users [21]. A fitness app, such as *Strava*, that rewards users for reaching fitness goals uses gamification. Game-based learning uses games to teach or train, but, unlike serious games, its main goal is not to achieve a learning outcome [22]. *Civilization* may be used by a history teacher to teach about historical events and civilizations, but it is not meant to teach history. Game-based learning uses games to teach or train without a specific goal.

#### Who? Serious Game Production Stakeholders

Different stakeholders from varying fields are involved in serious game production and development. Common stakeholders include the following [16]:

- Game developers are responsible for designing and developing the game. They create captivating game mechanics, visuals, audio, and more.
- Subject matter experts (SMEs) are knowledgeable about the serious game topic. They provide content and knowledge for game accuracy and effectiveness.
- Teachers and trainers can use serious games as a teaching or training tool for students or employees. They demonstrate how the serious game can meet learning goals.
- Game publishers distribute serious games to a wider audience. They market, distribute, and sell the game.
- Players are serious game consumers. They play the game and give feedback to improve it.
- Funders and sponsors are individuals or organizations that provide financial support for the development of the serious game (eg, government agencies, private foundations, or businesses).

#### When? Phases of Serious Game Production

Serious game production follows a similar process to traditional game development but with educational or training goals. The following are the five main serious game development phases:

1. The first step in serious game development is to identify the game’s learning objectives, audience, and context. This usually involves a needs assessment or curriculum analysis to identify gaps or areas where game-based learning could be beneficial [15].
2. A detailed plan for game mechanics, story, user interface, and learning content is created during the design phase. This phase may include storyboarding, prototyping, and playtesting to ensure that the game is engaging and meets learning objectives [16].
3. Development includes creating the serious game, including programming, artwork, audio, and multimedia assets. Game developers, instructional designers, SMEs, and other stakeholders work together to ensure that the game meets educational or training goals [23].
4. After creating the serious game, it is tested and evaluated to ensure that it meets its learning objectives. User testing, focus groups, and other student and stakeholder feedback methods may be used [24].
5. The serious game can be deployed for use in educational and training settings in the deployment and maintenance phase and should be supported and updated to ensure that it remains relevant and effective over time.

Serious game developers use the aforementioned steps to create outcome-aligned educational and training games. Such a framework supports agile game development and various development methods.

#### Where? TOGAF ADM

Serious games can make use of TOGAF ADM. Serious games with multiple stakeholders and complex systems require a structured EA development process. TOGAF ADM can also be customized for different industries and organizations. Such a broad view ensures that the game aligns with organizational goals, making it a good choice for serious game creators. TOGAF ADM is a nine-phase, sequential process for EA [25]:

1. Architecture vision: the EA team creates a high-level vision of the organization’s desired future architecture state. This phase determines the architecture development’s business drivers, stakeholders, and scope.
2. Business architecture: phase 2 involves understanding the organization’s business processes, objectives, and strategies. This phase produces business architecture artifacts that describe the organization’s business capabilities, value streams, and structure.
3. Information systems architecture: this phase focuses on understanding the organization’s information systems and technology infrastructure. This phase creates architecture artifacts for the organization’s application, data, and technology architecture.
4. Technology architecture: phase 4 focuses on selecting and defining technology components for implementing the organization’s architecture. This phase creates architecture artifacts for the organization’s technology infrastructure’s hardware, software, and networks.
5. Opportunities and solutions: phase 5 evaluates architecture solutions that meet business goals and objectives. This phase creates architecture artifacts that describe proposed solutions and their organizational impact.
6. Migration planning: in this phase, a plan is created to transition the organization’s architecture to its desired future state. This phase produces architecture artifacts that describe transition activity sequence and timing.
7. Implementation governance: phase 7 oversees the implementation of architecture solutions and ensures alignment with organizational objectives and goals. Governance framework architecture artifacts are produced in this phase.
8. Architecture change management: this phase manages ongoing changes to the organization’s architecture, aligning them with its goals and objectives. This phase produces architecture artifacts that describe change management.
9. Architecture evaluation: this phase evaluates the effectiveness of the architecture solutions and ensures that they meet the organization’s goals and objectives. This phase creates architecture artifacts that describe the evaluation process and results.

Interest groups can create EA solutions that meet their business goals by following TOGAF ADM. Moreover, TOGAF application to serious game development requires several crucial steps. First, an architecture vision is created to describe the game’s goals, objectives, target audience, and learning outcomes. Stakeholder analysis is then performed to identify the game development stakeholders and their needs and expectations. This ensures that the game is designed with stakeholders in mind. Architecture requirements describe the game’s functional and nonfunctional needs. The architecture development phase designs the game’s architecture based on the previous step’s requirements. This includes game mechanics, visual and audio assets, user interface, and layout design, that is, build, code, integrate visual and audio assets, and test the game’s usability and efficacy. To ensure stakeholder satisfaction, the game is monitored and evaluated over time. This may involve player feedback, game performance data analysis, and adjustments. Specifically, this work references the application of an ADM to serious game development and highlights how it can assist organizations in creating the desired strategic resource game. Doing so emphasizes that the ADM not only assists in the development of the serious game but also identifies organizational capabilities, methods, and processes that can be leveraged in future projects, thereby enhancing the team’s effectiveness.

#### Why? Stakeholder Identification, Analysis, and Management

Serious game design requires a stakeholder-centered conceptual framework for the following reasons:

- A stakeholder-centered approach considers various stakeholders’ needs and expectations during design. This may lead to more effective, engaging, and audience-relevant games (Bopp, J, A, unpublished data, December 2020).
- A stakeholder-centered approach ensures that the game is designed for the end user, improving usability and effectiveness. This improves player engagement, learning, and game performance [26].
- A stakeholder-centered approach can involve stakeholders in the design process, facilitating participation and acceptance. By increasing stakeholder confidence and ownership, serious game adoption and implementation can succeed [27].
- To improve serious game sustainability and scalability, a stakeholder-centered approach can be used to design games that meet the evolving needs of stakeholders. This can help the serious game stay relevant and effective as stakeholders’ needs change [28].

Serious game development relies on stakeholder identification, analysis, and management. This process begins with stakeholder identification. Stakeholder analysis prioritizes their needs and interests, while surveys, interviews, and focus groups help understand them. Stakeholder management involves planning how stakeholders will be engaged, their needs met, and their feedback incorporated into the serious game. Serious game development can use stakeholder management techniques such as regular meetings, an engagement plan, a registry, the prioritization of needs, feedback, and data analytics. Developers can create more effective, engaging, and audience-relevant games by managing stakeholder needs and expectations. A stakeholder-centered framework is needed for serious game design to ensure that stakeholders’ needs are met and to improve game effectiveness, usability, and sustainability.

#### How? Stakeholder Identification, Analysis, and Management

Stakeholder identification, analysis, and management are crucial to project success, including serious game development. Stakeholders are people or groups who care about the project’s outcomes and can influence them. Successful stakeholder identification, analysis, and management follow these four steps:

1. Identify internal, external, primary, and secondary stakeholders. Stakeholder analysis maps stakeholders and identifies key players [29].
2. Analyze stakeholders’ interests, needs, expectations, and influence on project outcomes. A matrix that maps stakeholders by power and interest can do this [30].
3. The project team can develop strategies to manage stakeholder relationships based on stakeholder analysis. This involves prioritizing stakeholders by influence and interest and creating stakeholder-specific engagement strategies [31].
4. Implement stakeholder management strategies through ongoing communication and engagement, such as project updates, meetings, and consultations. Stakeholder interests must be monitored and the stakeholder management plan adjusted [32].

These steps for identifying, analyzing, and managing stakeholders can help serious game developers maximize project success and build long-term relationships. When a new serious game project begins, the organization or team will already know the relevant stakeholders from stakeholder management and EA. Thus, each project improves the organization or team.

## Methods

### Framework Development

#### Overview

This section discusses how the integrative literature review revealed relevant theories, determined its limits, found relevant sources, collected terminology, defined its theoretical pillars, and provided practical approaches to the stakeholder-centered framework. Moreover, this section assesses the framework’s evolution over time; and it also theorizes future representations; reviews design processes; suggests improvements; and states the artifact design’s aggregate, iterative, and consistent impacts.

#### Variant 1: Informed by Literature

The preliminary snapshot of the stakeholder-centered framework is a compilation of ideas for a flexible, general-purpose framework to aid in the design of serious games. Initial concepts included generating, developing, and visually communicating the system’s fundamental elements, with a focus on user needs and empathy for the target demographic of serious game design stakeholders. Existing serious game literature, models, and approaches inform the framework variant, and an early exploration of these works provides a knowledge base for further consideration. Understanding the methods used in previous research on the same or similar issues assists in determining which methods will be most beneficial to advancing the topic and can aid in the evaluation of prior studies.

Various sources are represented in this formation because serious games’ content, definition, sources, liminal works, methods, and existing frameworks are investigated. As such, a substantial portion of this work is theoretical in nature and largely represents the efforts to seek and collect literature on the nature of serious games.

Textbox 1 shows how theoretical and experiential exploration shaped our initial project impression. First, because serious game projects require people and management, stakeholder theory was added to the framework. Second, early EA readings may help organizations achieve their goals. The framework’s third pillar, serious game design theory, positions the research and establishes its context. From this early stage, the framework must be applied and evaluated to determine its value for practitioners in the given milieu. This variant was extensively developer (self) reviewed. These steps close the DSR cycle loop and indicate that each variation is evaluated, even if reflectively.

**Sources that informed the first variant of the framework.**

**Sources and detail with explanation**

- Deterding et al [21]
  The authors define “gamefulness” and “gamification.” This influential work examines the differences between full-fledged games, serious games, pervasive games, extending games, game elements, and playful interaction. Even if not adopted, their definitions of “gamification” and “gamefulness” in contrast to serious games and playful interaction refine discourse and enable researchers to better understand and analyze the phenomena.
- Annetta [33]
  The author has presented a nested model of educational game design elements. Serious games have 6 elements, ranging from identity to instruction. This paradigm is hierarchical, with identity as the foundation for serious game design.
- Garris et al [23]
  A model by the authors shows the learning approach used in educational game research and its results. First, the main goal of any instructional content is to create a game-like educational program. Second, these qualities trigger a loop of user perceptions or responses such as interest or delight, user behaviors such as perseverance or concentration, and system input. If designers can match educational content with game elements, this cycle creates repeatable and self-motivated play. Third and last, game participation achieves training goals and learning outcomes.
- Ferdig [34]
  The authors define the “heart of serious game design” as theory, content, and game design. Serious game success requires emergent theory, content, and game design knowledge. Managing disciplinary conflicts and agreeing on serious game design is a major challenge for serious game teams.
- Marne et al [35]
  The authors list 6 serious game design aspects. This serious game design methodology shows the importance and distinction of pedagogical and game design expertise and their role in serious game development. This model’s main benefit is selecting the right experts for each design area.
- Rooney [36]
  The author proposes a triadic serious game design framework that considers pedagogy, play, and fidelity to create media.
- Vanden Abeele et al [37]
  The authors advocate the player-centered, iterative, interdisciplinary, and integrated (P-III) serious game design framework. This prominent framework provides a way for creating serious games that hinges on 4 conceptual pillars: player-centered design (from user testing during development to participatory design workshops during the design phase, projects start with inquiries that are influenced by ethnographic research), iterative development (the team establishes multiple milestones, and user testing culminates in a final prototype that can be evaluated), interdisciplinary teamwork (collaboration between instructional and game designers), and integration of play and learning (seamless blend between the game vision and core mechanics on the one hand and learning principles on the other hand).
- Yussof et al [38]
  The authors propose a serious game conceptual framework. The suggested outline combines gaming requirements with learning and pedagogy theory to provide a conceptual framework for serious game designers and educators.
- Gee and Hayes [39]
  The authors adapted the mechanics, dynamics, and aesthetics (MDA) framework into the design, play, and experience (DPE) framework. The extended DPE framework shows serious game layers for storytelling, learning, game play, and user experience. Every layer includes design, play, and experience.
- Roungas and Dalpiaz [40]
  The authors created a conceptual model of serious games to reduce misconceptions in serious game design teams by specifying a standard terminology that stakeholders can accept. The conceptual model also guides serious game design to address Game Design Document and other record keeping and administrative process inconsistency. Combining educational and game elements is the main challenge. Completed conceptual models are displayed in unified modeling language (UML) class diagrams.
- Breuer and Bente [41]
  The authors examine how serious games relate to e-learning and game-based learning. Serious games may use different learning strategies than edutainment and e-learning, according to them. According to the authors, many serious game definitions and typologies are limited.
- Ferdig [34], Rooney [36], and Deci and Ryan [42].
  Our novel synthesis combines the DPE framework, the serious game design framework proposed by Rooney [36], and self-determination theory (SDT). The idea emphasizes the importance of theory (pedagogy), content (fidelity), and game design (play) in serious game design. Effective serious game development is said to be central to these elements. In the DPE framework, SDT principles such as relatedness (the desire to connect with others), autonomy (the desire to choose one’s own paths), mastery (the desire to develop skills and master them), and purpose (the desire to connect actions with greater reason) are proposed to clarify or distinguish the connections between human psychological patterns and game features, mechanics, and dynamics to argue that gaming approaches and thinking can be successful. All 3 theories are combined to create a new serious game development strategy. The final stakeholder-centered framework partially incorporates these theories, but much of it leads the authors of this study to literature on game design.

#### Variant 2: Position, Activity, and Specialization

The next stakeholder-centered framework revisits unknowns and defines user problems to generate problem statements for subsequent design phases. Recordkeeping is stressed to avoid future issues. Stakeholder theory is emphasized, and how to identify and analyze serious game stakeholders is a key question. These stakeholders include experts, developers, and consumers, whose power and interest are analyzed using stakeholder analysis methods such as the power-interest grid. In the initial framework visualization, the EA pillar influences responsible, accountable, consulted, and informed matrices; Gantt charts; and business process model and notation swimlanes. In addition, variant 2 introduces 2 phases, idea validation and conceptualization, which continue in subsequent variants. We also discuss the 4 main serious game stakeholders from a previous stakeholder management approach: development team, publishers, context-related staff, and supplemental staff. Consumer stakeholders are consulted during development, but only the 3 (or 4) main categories are relevant to core game production.

As shown in Table 1, serious game production stakeholders often play multiple roles in smaller teams due to constraints. Variant 2 of the framework includes idea validation and conceptualization. The former evaluates the team’s serious game development prospects, while the latter starts project ideation. The framework has 3 levels: *execution*, *inquiry*, and *practical*. Serious game design stages include idea validation, conceptualization, development, and iteration in the execution level. Academic research and inquiry on serious game manufacture, participatory design, and more occur at the inquiry level. Stage-specific requirements and outcomes are listed in the practical level checklist.

**Figure 1:**
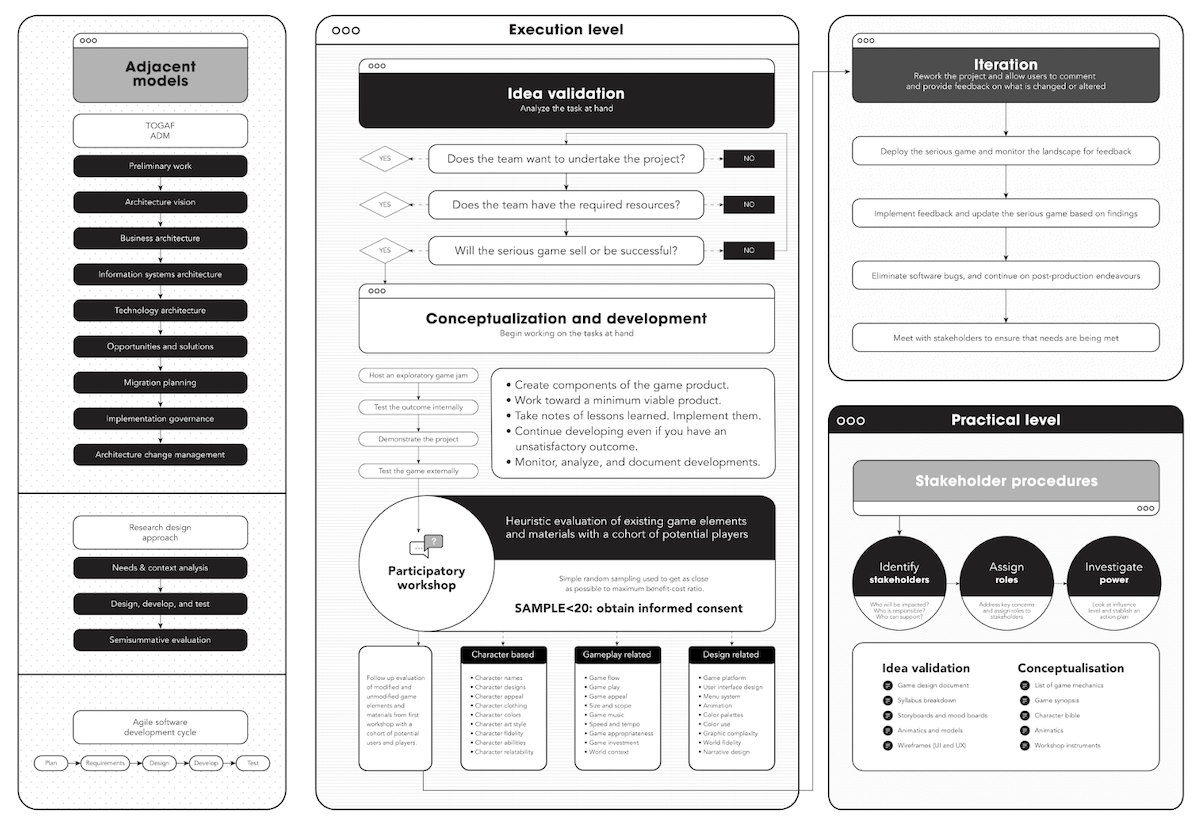
Simplified variant 2 of the stakeholder-centered framework. ADM: Architecture Development Method; TOGAF: The Open Group Architecture Framework; UI: user interface; UX: user experience. For a higher-resolution version of this figure, see Multimedia Appendix 1.

The 3 levels are necessary due to the complexity of serious game development. The variant 2 framework shown in Figure 1 [43] also includes TOGAF ADM, DSR design, and the agile software development life cycle. Collaboration, adaptability, and rapid prototyping are hallmarks of agile software development. Rapid prototyping, customer focus, flexibility, and serious game development improvement are promoted by this approach. Serious game development levels include TOGAF ADM, DSR design, and the agile software development life cycle.

**Table 1:** Serious game stakeholder categories, positions, activities, and specializations.

Category	Positions	Activities	Specializations
Development team	Programmer, artist, designer, producer, tester, composer, sound designer, and writer	The different tasks that game designers may perform during game development include coding, developing AI^a^ systems and camera systems, drawing characters and environments, designing UI^b^ elements, populating levels, managing the development team, managing schedules and resources, testing the game for bugs, creating music and sound effects, writing character dialogue, and setting up game objective prompts. Designers may also distinguish good from bad games and explain why, as well as ensure that the game achieves its goals and maintains its vision.	Over time, professionals specialize in 2D or 3D graphics, physics, mathematics, particle systems, UI, AI, input devices, and computer networking. Storyboard artist, concept artist, 3D modeler, environmental artist, texture artist, visual effects artist, UI artist, animator, technical artist, art director, level designer, game designer, system designer, scripter, combat designer, creative director, executive producer, associate producers, and assistant producers can specialize. Some positions are hired later in the development process and may be considered freelance rather than full time.
Publishing team	Product manager, project manager, creative manager, art director, technical director, marketer, and players and users	Publishing team members set priorities, review project milestones, set and meet targets or deadlines, provide feedback on improvements, collaborate with marketing to develop packaging and other visual assets, and promote the game. They also mediate between the studio and the publisher’s legal department, work with licensors and the ESRB^c^ to secure a rating, and provide technical support. They may also cooperate with marketing and PR^d^ on press materials, co-design the game, and improve its visual language.	These positions may or may not affect the game’s content and aim to streamline development and maintain quality within budget and time constraints. Game designers or writers in publishing usually fill these positions, which may vary in involvement depending on the publisher. In addition to programming, they may handle management issues and work with media outlets and advertising firms with different needs and capabilities.
Context-related team	Subject matter expert, educational theorist, scholar, and research director	They consult with teams on game content and requirements, provide educational material, maintain educational aspects, investigate and test game features, and manage or supervise scholars and data collection. They also propose, choose methods, supervise, budget, and report.	In all serious game design projects, these stakeholders must provide sufficient materials to address serious issues and express them through gameplay. Although they may not be educators, they focus on game curriculum and syllabus development. They may be specialists in a research field or pursuing specialization.
Supplementary team	Business developer, lawyer, brand manager, PR manager, quality assurance manager, talent recruiter, human resource officer, game reviewer, licensor, and funding entity	They create business opportunities, secure funding, and invest in games. They also provide legal advice, review contracts, and handle licensor negotiations. In addition, they maintain brand representation in the game and work with marketing on packaging, create marketing strategies, contact gaming publications and blogs, and organize press events. Moreover, they manage the test department, send developers bug sheets, and ensure quality, as well as recruit, manage, and train new hires.	Their responsibilities include building relationships with teams, reviewing game demonstrations, negotiating contracts, generating marketing strategies, managing the employment process, playing and reviewing games, and securing funding for serious game projects. Game producers run the test department, organize press events, recruit talent, and invest in serious game projects. These people play games, write reviews, and suggest improvements. In addition, they license IP^e^ and may work with licensors to get ratings. Moreover, they financially support serious game projects and crowdfund.

^a^AI: artificial intelligence.

^b^UI: user interface.

^c^ESRB: Entertainment Software Rating Board.

^d^PR: public relations.

^e^IP: intellectual property.

#### Variant 3: Refinement

Variant 3 of the framework emphasizes human-centeredness and separates idea validation and evaluation (Table 2). The 3 levels are *EA*, *execution*, and *practical*. Each level combines serious game and stakeholder theory literature, practices, and methods, but only the EA level fully represents 1 of the 3 TOGAF ADM research pillars. The levels help practitioners avoid not seeing the forest for the trees and understand the framework’s many components, mechanisms, and prescriptions. The composite nature of serious game development makes the framework stratiform, and the levels isolate and aggregate all interaction-based components, connectors, and relations for every aspect of the system’s functionality into a single structural model.

**Table 2:** The third framework variant.

EA^a^ level		Execution level	Practical level
This level establishes the strategic framework for serious game design, aligning game objectives with organizational goals and stakeholder needs through a comprehensive stakeholder analysis.		It transitions the process from strategic planning to the tangible design and development phases, detailing the game’s mechanics, story, and technical requirements to ensure alignment with the defined objectives.	This final level focuses on the deployment, testing, and evaluation of the game in real-world scenarios, emphasizing the adjustment of the game design based on user feedback and the effectiveness of achieving intended outcomes.
**Core functions**			
	This level focuses on the strategic aspects of serious game design, aligning game objectives with broader organizational or project goals. It incorporates stakeholder analysis to ensure that the game’s objectives meet the needs and expectations of all relevant parties.	At this level, the framework transitions from strategic planning to operational design and development. It involves the detailed design of the game, including gameplay mechanics, narrative elements, and technical specifications. This level ensures that the game’s design is feasible and aligns with the strategic objectives outlined at the EA level.	The practical level is where the game is deployed and assessed in real-world settings. This involves testing, gathering feedback from end users and stakeholders, and iterating on the design based on this feedback. The focus here is on practical application and the effectiveness of the game in achieving its intended outcomes. The level offers options for game developers: Prediscovery stage Basic: stakeholder team selection, assembled team, and game design document Standard: basic outputs, selection of game mechanics, and storyboards Advanced: standard outputs and detailed curriculum itemization Production Basic: stakeholder prioritization, game synopsis, and character bible Standard: basic outputs and mood boards Advanced: standard outputs, wireframes, and animatics Periphery Basic: Ongoing stakeholder prioritization, game art development, and level design Standard: basic outputs and prototype development Advanced: standard outputs, deeper design practice, and quality assurance

^a^EA: enterprise architecture.

### Ethical Considerations

This study was approved by the Institutional Review Board of North-West University, ensuring adherence to ethical standards in research involving human participants (approval number: NWU-01775-20-A9). Informed consent was obtained from all participants prior to their inclusion in the study (in the expert review questionnaire). Participants were informed of their right to opt out at any time without any consequences. Data collected during this study were anonymized to protect participant confidentiality. Identifiable information was removed, and data were stored securely in a password-protected database. Participants were not compensated for their time and effort in participating in the study. They were, however, promised a copy of the academic work once published.

## Results

### Expert Review Analysis

#### Overview

The expert review questionnaire regarding the stakeholder-centered framework was distributed to 220 serious game practitioners and experts internationally, of whom 29 (13.2%) completed it. On average, questionnaire completion took 57.2 (range 14.5-252) minutes. Considering the in-depth nature of the research, a completion time of approximately 24 minutes (excluding the outlier) is acceptable, despite the recommended 15-minute length for questionnaires. This study’s niche focus on serious games results in a smaller expert pool; thus, the response rate and data volume are considered satisfactory. The questionnaire, designed for comprehensive data collection on the stakeholder-centered framework, uses both qualitative and inferential statistical analyses.

The research accounts for web-based survey challenges by ensuring content validity and question clarity, balancing open-ended and closed-ended questions, and maintaining reliability. Despite initial plans, level-specific explainer videos were excluded to prevent extending the questionnaire’s length. An introductory explainer video was provided [44], and participants had full access to the framework for a thorough review. To ensure depth and accuracy, participants were granted access to all aspects of the framework to ensure that they could perform a multifaceted expert review.

#### Section A: General Information

The questionnaire respondents predominantly skewed younger, with 45% (13/29) aged 26 to 35 years and 38% (11/29) aged 36 to 45 years. Those aged 46 to 55 years constituted 10% (3/29) of the sample, while those aged 18 to 25 years and ≥66 years each represented 3% (1/29). White individuals made up 69% (20/29) of the respondents, followed by 14% (4/29) of individuals of other ethnic backgrounds including Hispanic individuals, Latinx individuals, people of color, and others. Asian respondents accounted for 7% (2/29) of the sample; and African, Indian, and undisclosed categories each accounted for 3% (1/29). Gender distribution among the respondents was fairly even, with 56% (16/29) identifying as man and 41% (12/29) as woman; of the 29 respondents, 1 (3%) preferred not to disclose their gender. In terms of geography, 45% (14/29) of the participants were from South Africa, reflecting the study’s origin and local interest. Thailand and Australia each contributed 7% (2/29) of the respondents, while the remaining countries (11/13, 85%) each contributed 3% (1/29) of the respondents, broadening the international representation.

#### Section B: Game Development Experience

##### Overview

The survey section on game design experience collected data on qualifications, occupations, and development experience, including roles and satisfaction in game development. The respondents had high qualifications, with 55% (16/29) holding doctoral degrees, 24% (7/29) master’s degrees, and 14% (4/29) honors degrees. The occupational profile was academic-centric, with 24% (7/29) being lecturers and 17% (5/29) senior lecturers. Others (17/29, 59%) included professors, researchers, and various roles in private industry. A significant proportion of the respondents (16/29, 56%) had >5 years of game development experience, showcasing their expertise in the field. Most (22/29, 76%) had also been involved in serious game development, although a few (3/29, 10%) had not, and some (2/29, 7%) were unsure or had projects in development. In terms of team experience, the majority (22/29, 76%) affirmed involvement, with a small percentage (3/29, 10%) either working independently or not at all in serious game development. Satisfaction across 11 development factors (DFs) was measured, with the highest scores (out of 4) being for collaboration (3.54), skills (3.56), vision (3.56), and educational aspects (3.7) and the lowest for management (2.85). The DFs and their resulting scores were as follows:

- DF1: collaboration, average score=3.54
- DF2: communication, average score=3.22
- DF3: resources, average score=3.19
- DF4: team composition, average score=3.3
- DF5: skills, average score=3.56
- DF6: management, average score=2.85 (this is the lowest average score among the 11 DFs studied)
- DF7: vision, average score=3.56
- DF8: procedures and processes, average score=2.96
- DF9: outcomes, average score=3.12
- DF10: conflict, average score=3.31
- DF11: educational and edifying aspects, average score=3.7 (this is the highest average score among the 11 DFs investigated)

Respondents expressed their views on various aspects of serious game development in this section:

- Collaboration (DF1): respondents were largely satisfied with their collaborative efforts in developing games.
- Communication (DF2): although rated slightly lower than collaboration, communication during serious game design was still positively regarded.
- Resources (DF3): the resources available for serious game development, including educational materials, software tools, and marketing aids, were deemed satisfactory.
- Team composition (DF4): the composition of serious game teams was viewed favorably, with the right mix of skills and expertise viewed as to the team’s goals and performance.
- Skills (DF5): team members’ skills were rated as fitting for serious game development tasks.
- Management (DF6): satisfaction with management was moderate, indicating that some areas may require improvement.
- Vision (DF7): respondents were content with the guiding visions for serious game projects, which help in goal setting and decision-making.
- Procedures and processes (DF8): there was some dissatisfaction with the processes involved in transforming ideas into final products.
- Outcomes (DF9): the outcomes of serious game projects were generally met with approval, suggesting satisfaction with the services or interventions provided.
- Conflict (DF10): opinions on conflict were mixed but leaned toward satisfaction with handling disagreements during serious game projects.
- Educational and edifying aspects (DF11): given the respondents’ backgrounds in education and research, they highly rated the educational value of the games produced.

The section B responses indicated that the experts were well-versed in game development, with a specific focus on serious game development. Their moderate satisfaction across key production factors attested to their practical experience, reinforcing the study’s credibility and reliability. Predominantly researchers, these individuals engage deeply with the field, often acting as SMEs in serious game projects. The most frequently reported challenges were resource-related: time, budget, and skills. Acknowledging these common hurdles faced by serious game professionals helps refine the framework to address and mitigate such issues more effectively.

##### Serious Game Development Roles

*Researcher* emerged as the most common role among serious game professionals, accounting for 14% (4/29) of the respondents, highlighting their involvement in data collection, analysis, and contribution to scholarly literature. The role of *educational theorist* followed at 10% (3/29), underscoring expertise in teaching methods. *Content expert*, *designer*, and *tester* each constituted 9% (3/29) of the respondents. Additional roles such as *project manager*, *CEO* (chief executive officer), and *UX* (user experience)/*UI* (user interface) designer were specified under *Other*. With education-related roles being predominant, this reflects the survey’s findings on respondent occupations. A total of 176 roles were reported, averaging 6 roles per person, indicating the multifaceted nature of serious game stakeholder involvement. The diversity of roles suggests that stakeholders often wear multiple hats in their projects. Notably, *lawyer* and *licensor* were the only roles not represented among the respondents.

##### Serious Game Development Activities

Respondents reported a broad spectrum of activities within serious game development, categorized into *preactivity stage*, *development*, *postactivity stage*, *continuous*, and *unknown*:

- *Preactivities* are preparatory steps such as topic research, fundraising, context analysis, problem definition, game scope determination, learning content creation, and initial consultations.
- *Development* activities encompass the actual creation process, including game design, iteration, implementation, programming, artwork, and character design.
- *Postactivities* might consist of usability testing and game evaluations, depending on the project’s goals.
- *Continuous* activities are ongoing tasks such as management, research, education, administration, and marketing that span the project’s life cycle.
- *Unknown* captures any unclear or undefined responses.

The bulk of the feedback pertained to the hands-on development tasks—programming, art, writing, and design—aligning with the framework’s emphasis on development processes. Game research and evaluation were equally represented, each with 9 mentions, while learning content development received 7 mentions, reflecting the educational aspect of serious game projects.

##### Serious Game Development Issues

Respondents were asked about common issues encountered during game development, with the question focused on *resources* and *game-specific* challenges.

Resource-related issues highlighted included the following:

- Time management, with 7 (24%) of the 29 respondents noting the extensive duration needed for serious game projects, often described as time consuming and unrealistic
- Budget constraints, also mentioned by 7 (24%) of the 29 respondents, indicating that limited funding, especially within educational environments, affects the scope of development
- Skills shortage, with responses pointing to a lack of necessary expertise and experience among serious game stakeholders
- Team-related factors, with, for example, size and composition, tools for development, intellectual property concerns, and marketing resources highlighted as challenges

Game-specific issues centered on the following aspects:

- The balance between educational content and entertainment value, with respondents expressing difficulty in finding the right mix
- Validation of serious game effectiveness, including measuring the impact of serious games on players, which was mentioned as a key concern

End-user considerations include player demographics, abilities, and gaming background, along with their engagement levels and ability to reach states of flow during gameplay.

#### Sections C, D, and E: Framework-Level Impressions

The 3 levels of the framework are *EA*, *execution* (process oriented), and *practical* (outcomes). The following aspects of the conceptual framework levels were examined in sections C, D, and E of the expert review questionnaire:

- Comprehensibility: the degree to which the framework, including its overall structure and key components, can be understood and comprehended by its intended audience
- Fluency: the ease with which the framework can be applied or implemented by its users, considering the clarity of instructions and the usability of any associated tools and resources
- Length: the appropriate duration or scope of the framework to ensure that it is neither too long nor too short and provides adequate guidance to achieve the desired results
- Accessibility: the extent to which the framework is accessible to all potential users, including those with physical or cognitive limitations, and the availability of the resources required to implement the framework
- Applicability: the relevance and utility of the framework in addressing the challenges or opportunities it is intended to address
- Utility: the effectiveness of the framework in achieving its intended outcomes, including its capacity to produce measurable and quantifiable outcomes
- Contextuality: the extent to which the framework is tailored to the context or situation in which it will be applied, including cultural and social considerations
- Outputs: the tangible and measurable results or outcomes produced by the application of the framework, such as changes in behavior and performance enhancements, as well as other demonstrable effects

The results from sections C, D, and E are presented in Table 3.

**Table 3:** Questionnaire results for sections C, D, and E (n=29).

Framework aspect		5-point scale ratings						Overall comments
		Strongly agree, n (%)	Agree, n (%)	Neutral, n (%)	Disagree, n (%)	Strongly disagree, n (%)		
**Comprehensibility (clarity of framework structure and components)**								
	EA^a^ level	—^b^	9 (33)	11 (41)	—	7 (26)	Needs more clarity	
	Execution level	—	21 (75)	6 (21)	—	1 (4)	Clear to most respondents	
	Practical level	23 (81)	—	—	—	—	Rated highest for clarity	
**Fluency (ease of applying or implementing the framework)**								
	EA level	19 (68)	—	8 (29)	—	1 (4)	Logical progression noted	
	Execution level	22 (79)	—	4 (14)	—	2 (7)	Highly logical flow	
	Practical level	23 (81)	—	—	—	—	Streamlined progression	
**Length (adequacy of framework duration and scope)**								
	EA level	—	7 (27)	13 (46)	7 (27)	—	Divided opinions	
	Execution level	13 (46)	—	11 (39)	4 (14)	—	Some found it lengthy	
	Practical level	17 (62)	—	—	—	—	Most agreeable length	
**Accessibility (ease of access for all users, including those with limitations)**								
	EA level	—	5 (20)	12 (44)	10 (36)	—	Complex for laypersons	
	Execution level	—	13 (48)	11 (41)	3 (11)	—	Accessible to general users	
	Practical level	13 (48)	—	—	—	—	Most accessible level	
**Applicability (relevance and adaptability of the framework to different scenarios)**								
	EA level	—	11 (39)	13 (48)	3 (11)	—	Requires more relevance	
	Execution level	19 (68)	—	7 (25)	2 (7)	—	Pertinent to game design	
	Practical level	21 (74)	—	—	—	—	Highly relevant to practice	
**Utility (effectiveness in producing intended outcomes)**								
	EA level	—	10 (36)	15 (54)	3 (11)	—	Utility is acknowledged	
	Execution level	—	10 (36)	15 (54)	3 (11)	—	Comparable to EA level	
	Practical level	13 (46)	—	13 (46)	—	—	Divided on effectiveness	
**Contextuality (suitability of the framework for various cultural and social settings)**								
	EA level	—	10 (36)	15 (52)	3 (11)	—	Needs more adaptability	
	Execution level	15 (52)	—	7 (26)	3 (11)	—	Flexible across settings	
	Practical level	13 (48)	—	—	—	—	Well-tailored to contexts	
**Outputs (tangible results or benefits from using the framework)**								
	EA level	—	10 (37)	13 (48)	4 (15)	—	Essential outputs produced	
	Execution level	16 (57)	—	9 (32)	3 (11)	—	Effective in generating outcomes	
	Practical level	19 (67)	—	—	—	—	Highest positive review	

^a^EA: enterprise architecture.

^b^Not applicable.

#### Section F: Overall Impressions

Expert feedback on the stakeholder-centered framework revealed several key themes. Most of the participants agreed that the framework is useful for facilitating serious game development, highlighting its organized approach and detailed guidance. However, worries about its intricacy indicate that it could be overwhelming for smaller teams or individuals inexperienced in serious game development. Some of the respondents proposed that the framework should be used primarily as a diagnostic tool rather than a prescriptive one, hinting at a possible adjustment in its application to better suit serious game developers’ varying levels of expertise.

Feedback on the framework’s features suggested the necessity for additional refinement to improve its structure and comprehensibility. Respondents requested a clearer definition of serious game design elements and a greater emphasis on the gameplay experience. The relationship between the different levels of the framework and how well they work together with other frameworks were recognized as areas that require focus. There was a clear desire for participatory processes, emphasizing a preference for a stakeholder-centered approach that maximizes production and team engagement.

The framework’s implementation elicited varied responses, with a significant portion finding it easy to use, while a noteworthy percentage encountered challenges. These observations emphasize the significance of customized training and emphasize the intricate nature of the framework. Providing stakeholders with necessary tools and ensuring that the framework is easily accessible can help alleviate these challenges.

Opinions on the provision of necessary development instruments were mostly positive, although some of the respondents noted that the framework does not fully address all aspects of information systems development or early serious game design requirements. This indicates potential for growth and a more detailed incorporation of serious game design mechanisms. Many of the respondents viewed the motivation to excel while using the framework positively because it offers clarity on roles and progression through the serious game development stages. However, some of the respondents doubted its impact on motivation, citing the possibility of heightened demands because of the framework’s procedural intricacy. Respondents had a positive outlook on how the framework would affect stakeholder efficiency and management, expecting enhancements in planning and stakeholder engagement. However, some of them believed that it might lead to increased resource demands and project delays, highlighting the importance of finding a balance between specific instructions and managing work efficiently.

### Summary

Overall, the stakeholder-centered framework was acknowledged as a valuable tool for serious game development, but it requires simplification and more user-friendly adjustments. The feedback is crucial for future improvements, guaranteeing that the framework stays pertinent and efficient for various serious game development scenarios.

## Discussion

### Principal Findings

**Figure 2:**
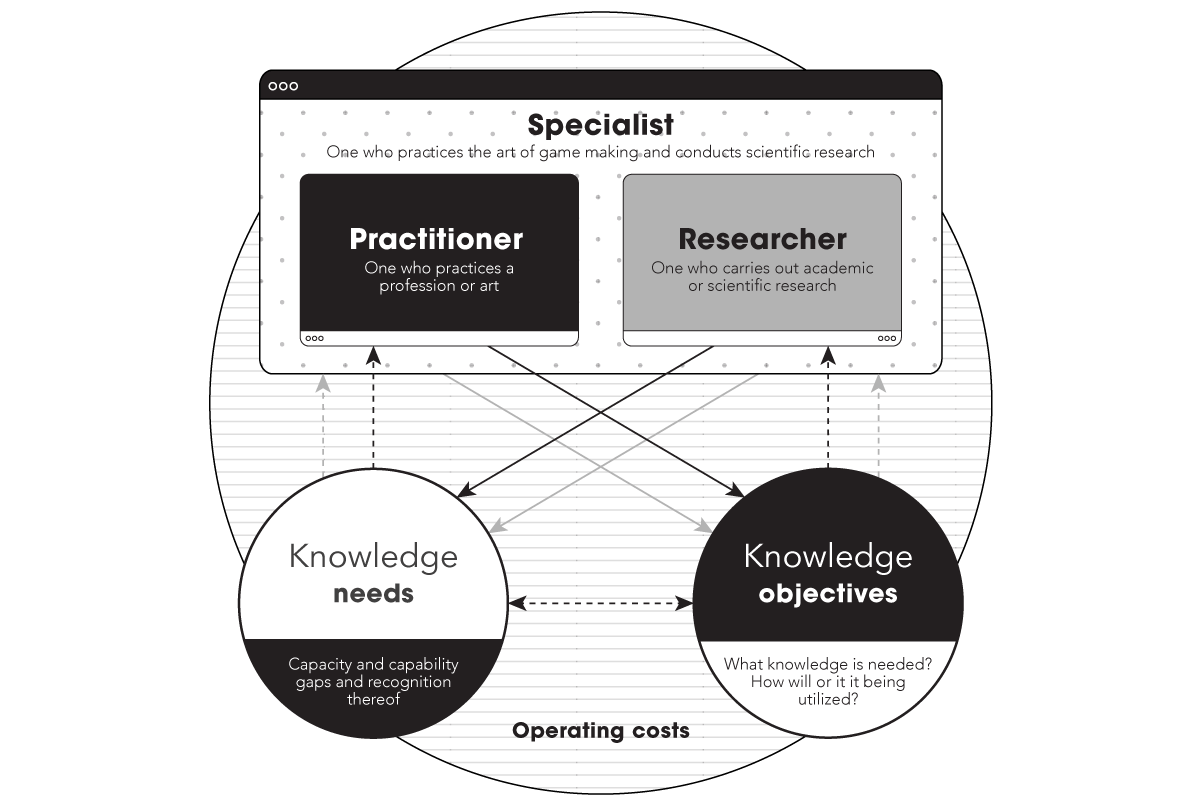
Overcoming variance in knowledge needs and objectives for serious game practitioners, researchers, and specialists.

The study highlights that serious game practitioners, researchers, and specialists have varying knowledge needs and objectives; for instance, a serious game practitioner in private industry may seek financial information related to serious games for profitability, while a researcher may focus on evaluating the effectiveness of serious game media. Serious game specialists may require a combination of knowledge needs to fulfil their role. As indicated in Figure 2, it is crucial for managerial staff to be aware of these differences and understand their team members’ knowledge needs and objectives to effectively manage operating costs and stakeholder needs in a serious game project.

The neglect of elements such as threat assessment in serious game practice can lead to increased risk, which can impact revenue and project success. It is important for administrative and managerial staff to consider different types of risks, such as integrated, behavioral, strategic, financial, compliance, legal, and operational risks before, during, and after serious game production. Risk assessment has significant implications for stakeholder management, uncertainty management, hazard evaluation, control measures, and workplace safety and should be considered in any framework aimed at supporting stakeholders in creating effective serious game media.

The stakeholder-centered framework is mostly prescriptive and lacks personalization, and stakeholder input is necessary for improvement. The framework also lacks emphasis on game design principles and evaluation. Future research can explore ways to facilitate stakeholder participation and integrate *serious* considerations such as learning analytics, knowledge management systems, evaluation frameworks, and more.

### Limitations

One limitation is the challenge of incorporating 3 different theoretical pillars, which increases the complexity and can make it difficult to achieve brevity in practical research. In addition, the qualitative nature of the investigation means that the focus is on a specific sample group with distinct demographic, psychological, social, and cultural traits, making it difficult to generalize the findings to all comparable groups or circumstances. As a result, transferability is more relevant for this qualitative research.

Time constraints are another limitation imposed on the study because it is cross-sectional in nature, giving us a limited time frame to deliver our work for examination. However, the DSR approach allows the framework to undergo imminent development and iteration.

The modest sample size of the study (n=29) could also be considered a limitation because serious games are a niche field that often require expertise in education, health, or public policy, which may limit their developers. However, we contacted 220 people to take part in the study after extensive market research, and additional data would have reduced random variation and increased statistical power, making the research more accurate and reliable.

Another limitation of the research is that the framework could only be evaluated on a particular level of response assessment. Future studies should be conducted with teams to see how the framework functions in practice, according to all 4 levels of artifact assessment: response (participant feelings), learning (knowledge transfer), behavior (work performance), and outcomes (effect over time).

Finally, the reliability of questionnaire data analysis is highly dependent on several factors, such as the quality and depth of the responses, the structure of the questionnaire, and the lack of observations regarding alterations in the respondents’ states of mind, feelings, and behaviors. Therefore, these factors should be taken into consideration when interpreting the results of the study.

### Recommendations

We offer the following recommendations for future work:

- Forthcoming work on this topic should isolate each theoretical domain, examine them discretely, and combine, compare, and synthesize the results.
- A positivistic study that gathers quantitative data would intensify the generalizability of the findings relating to the stakeholder-centered framework. Quantitative research, such as experimental studies, offers a good basis for developing wide generalizability, given that generalizability requires data from large populations.
- Longitudinal research over an extended period of time could better assess the affects and effects of the framework. The analysis could also be richer if the inquiry extends beyond a single moment in time. DSR is typically carried out in iterative cycles of design, implementation, and evaluation, which enables researchers to refine and improve their solutions over time, allowing for strong longitudinal studies. This iterative approach also allows for data collection at multiple points in time, which can provide insights into the effectiveness and long-term viability of the solutions being developed. The framework becomes a living artifact in this way.
- A larger sample population can feature in impending studies. The greater the sample size, the more precise the calculated mean values will be. Error margins are also reduced if a bigger sample is used.
- More participants enable the facts to speak for themselves, rather than depending on assumptions and the researcher’s subjective relationships with the data. Additional data also lead to more accurate and precise units of analysis.
- The stakeholder-centered framework can be assessed according to all 4 levels of artifact assessment proposed by Petri and von Wangenheim [45]. A longitudinal study of this nature would be equipped to establish the effectiveness of the framework regarding its learning potential, behavioral impacts, and outcomes.
- The methods used to appraise the framework could be expanded in future work. This would improve the reliability of the data analysis carried out; for example, structured interviews, semistructured interviews, in-depth interviews, focus groups, field research, ethnography, and observation could be used to strengthen analysis efforts.

### Features for the Framework Going Forward

Now that that main recommendations for future research have been presented, we need to consider the requisite features for the framework going forward (Table 4).

**Figure 3:**
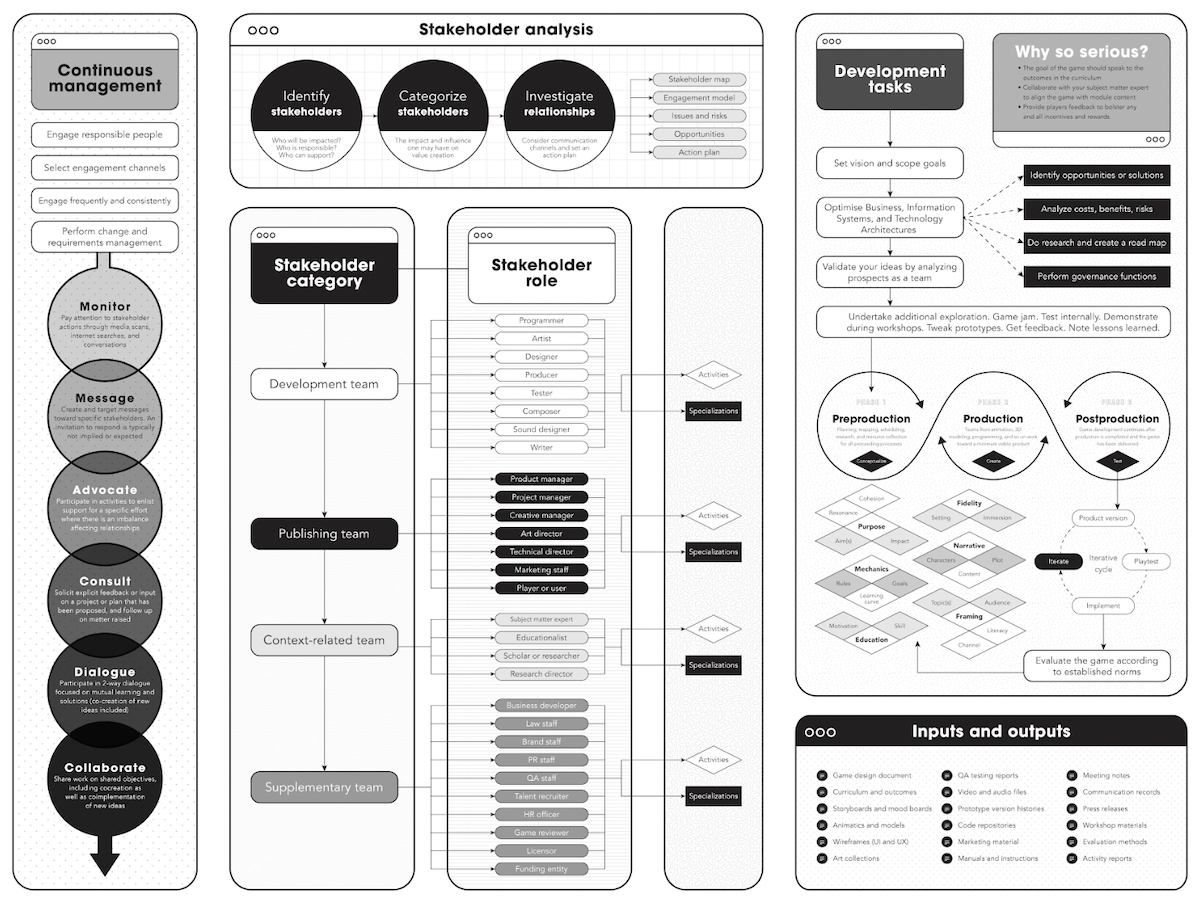
Variant 4 (version 2.0) of the stakeholder-centered framework. HR: human resources; MVP: minimum viable product; PR: public relations; QA: quality assurance; SME: subject matter expert; UI: user interface; UX: user experience. For a higher-resolution version of this figure, see Multimedia Appendix 2.

Variant 4 of the stakeholder-centered framework reflects the academic and investigative nature of serious games and their development (Figure 3). It includes instructions, demonstrations, descriptions, and definitions that facilitate game design and development and focuses on procedural information rather than technology, expertise, or resources. The length of the tool has been reduced, and the framework is less prescriptive, providing flexibility in project assumptions, goals, and processes. The progressive web application version of the framework enables users to take part in conversations with one another, categorizes procedural information into subdivisions, and includes built-in support mechanisms. The application affords the researcher added control over the transmission, presentation, structure, and extent of the intelligent system and can adapt to any changing needs or patterns of its user base.

Variant 4 also integrates the 3 theoretical domains in a more subtle manner than previous versions. It presents a terser technical diagram for serious game design that omits some information to improve accessibility and usability. The procedures still begin with stakeholder analysis, categorizing stakeholders by their impact and influence on value creation in the development endeavors. The resulting stakeholder categories are development, publishing, context-related, and supplementary teams, which consist of stakeholder roles with their own activities and specializations.

**Table 4:** How desired framework traits correlate to improvement areas, as well as evidence for the intersection thereof in the progressive web artifact.

Desired framework trait	Improvement areas (from expert review)											Artifact execution (within the progressive web application)
	Abbreviation	Concentration	Configuration	Guidance	Idealism	Milestones	Prescription	Semantics	Simplification	Transmission		
Concerned with serious aspects		✓		✓			✓	✓			Serious game conventions built into the artifact: principles, designs, and evaluations	
Concise	✓		✓					✓	✓	✓	Artifact uses chunking to boost content processing	
Diagnostic		✓		✓		✓				✓	Artifact facilitates the achievement of development objectives through measurement	
Flexible	✓		✓	✓			✓		✓	✓	Artifact is open to changes in assumptions, goals, and process	
Informative		✓		✓	✓	✓		✓			Artifact includes instructions, demonstrations, descriptions, and definitions on serious game design	
Invested in work, not technology		✓	✓	✓				✓		✓	Artifact makes provision for technology but focuses on procedural information	
Participatory		✓	✓				✓			✓	Discussion is facilitated in the artifact by way of private and community chat functions	
Procedural			✓	✓	✓	✓	✓			✓	Various activities are divided into practical sections and directed at various stakeholders	
Relevant		✓		✓				✓			Extraneous information is removed using filters, and streams of material are categorized	
Repeatable	✓			✓		✓			✓	✓	Sets of actions provided in the artifact are reusable and easily duplicated	
Stakeholder centered		✓			✓		✓			✓	Sections of the artifact are targeted toward specific stakeholders	
Supportive			✓	✓		✓	✓		✓		Frequently asked questions, data protection, and self or continual support	
Sustainable	✓			✓		✓				✓	Scalable design is incorporated into the artifact	
Usable	✓	✓	✓	✓	✓	✓	✓	✓	✓	✓	User control, consistency standards, minimalist design, and platform compatibility	
Vehicle for good design		✓	✓			✓	✓			✓	Artifact encourages good design practice by encouraging users to assess their own practice	

### Conclusions

The proliferation of serious games and game-assisted learning in education and beyond requires keen awareness, careful contemplation, and steady advancement [46-48]. As serious games become more common in contexts aiming to edify in innovative ways, scholars should not only consider methods to improve the efficacy thereof but also think about how to realistically and efficiently fabricate them as well. Serious game project stakeholders need practical ways to align their interests with those of the enterprise. Stakeholder roles, activities, specializations, potential, competence, and capabilities all impact these experts’ productive capacity and labor prospects. However, serious game initiatives vary significantly. To help future game makers, this research inspects serious game design stakeholders and techniques to produce a system capable of supporting these individuals in a range of environments. A stakeholder-centered framework, in this view, may help serious game developers manage their teams and drive practice in beneficial and sustainable ways. In the future, we hope that this investigation will aid in a decrease in serious game project failure, communication breakdown, and apathy regarding the genre of games intending to do more than purely entertain. However, additional research and innovation is needed in fields adjacent to, and embedded in, serious games to support the growing need for novel approaches to demonstrate, educate, simulate, and inform.

## Appendix

Multimedia Appendix 1 Simplified variant 2 of the stakeholder-centered framework. ADM: Architecture Development Method; TOGAF: The Open Group Architecture Framework; UI: user interface; UX: user experience.

Multimedia Appendix 2 Variant 4 (version 2.0) of the stakeholder-centered framework. HR: human resources; MVP: minimum viable product; PR: public relations; QA: quality assurance; SME: subject matter expert; UI: user interface; UX: user experience.

## Abbreviations

ADM: Architecture Development Method
CEO: chief executive officer
DF: development factor
DSR: design science research
EA: enterprise architecture
SME: subject matter expert
TOGAF: The Open Group Architecture Framework
UI: user interface
UX: user experience

## Data Availability

The data sets generated and analyzed during this study are available from the corresponding author on reasonable request.

## References

[1] Girard C, Ecalle J, Magnan A (2012). Serious games as new educational tools: how effective are they? A meta‐analysis of recent studies. Comput Assist Learn.

[2] Spil TA, Romijnders V, Sundaram D, Wickramasinghe N, Kijl B (2021). Are serious games too serious? Diffusion of wearable technologies and the creation of a diffusion of serious games model. Int J Inf Manage.

[3] Clapper TC (2018). Serious games are not all serious. Simul Gaming.

[4] Suryapranata LK, Soewito B, Kusuma GP, Gaol FL, Warnars HL (2017). Quality measurement for serious games. Proceedings of the International Conference on Applied Computer and Communication Technologies (ComCom).

[5] Pacheco-Velazquez E, Bester A, Rabago-Mayer L, Ro V (2023). What do we evaluate in serious games? A systematic review. Proceedings of the 17th European Conference on Games Based Learning.

[6] Koscianski A, Zanotto D (2014). A design model for educational multimedia software. Creat Educ.

[7] Taylor L (2004). Video game internal turfs and turfs of play. M/C J.

[8] Gaspar JD, Lage EM, Silva FJ, Mineiro É, Oliveira IJ, Oliveira I, Souza RG, Gusmão JR, De Souza CF, Reis ZS (2020). A mobile serious game about the pandemic (COVID-19 - did you know?): design and evaluation study. JMIR Serious Games.

[9] Blakey H (2021). Designing player intent through “playful” interaction: a case study of techniques in transistor and journey. M/C J.

[10] Landers RN (2015). Developing a theory of gamified learning: linking serious games and gamification of learning. Simul Gaming.

[11] Haoran G, Bazakidi E, Zary N (2019). Serious games in health professions education: review of trends and learning efficacy. Yearb Med Inform.

[12] Carlier S, Van der Paelt S, Ongenae F, De Backere F, De Turck F (2020). Empowering children with ASD and their parents: design of a serious game for anxiety and stress reduction. Sensors (Basel).

[13] Tan JW, Zary N (2019). Diagnostic markers of user experience, play, and learning for digital serious games: a conceptual framework study. JMIR Serious Games.

[14] Scurati GW, Nylander JW, Ferrise F, Bertoni M (2022). Sustainability awareness in engineering design through serious gaming. Des Sci.

[15] Alvarez J, Michaud L (2008). Serious Games : Advergaming, Edugaming, Training.

[16] Connolly TM, Boyle EA, MacArthur E, Hainey T, Boyle JM (2012). A systematic literature review of empirical evidence on computer games and serious games. Comput Educ.

[17] Sitzmann T (2011). A meta‐analytic examination of the instructional effectiveness of computer‐based simulation games. Pers Psychol.

[18] Baranowski T, Blumberg F, Buday R, DeSmet A, Fiellin LE, Green CS, Kato PM, Lu AS, Maloney AE, Mellecker R, Morrill BA, Peng W, Shegog R, Simons M, Staiano AE, Thompson D, Young K (2016). Games for health for children-current status and needed research. Games Health J.

[19] Bogost I (2007). Persuasive Games: The Expressive Power of Videogames.

[20] Michael DR, Chen · S (2006). Serious Games: Games that Educate, Train and Inform.

[21] Deterding S, Dixon D, Khaled R, Nacke L (2011). From game design elements to gamefulness: defining "gamification". Proceedings of the 15th International Academic MindTrek Conference: Envisioning Future Media Environments.

[22] Plass JL, Homer BD, Kinzer CK (2016). Foundations of game-based learning. Educ Psychol.

[23] Garris R, Ahlers R, Driskell JE (2016). Games, motivation, and learning: a research and practice model. Simul Gaming.

[24] Kato PM (2010). Video games in health care: closing the gap. Rev Gen Psychol.

[25] (2018). The TOGAF ® Standard, Version 9.2.

[26] Kato PM (2012). Evaluating efficacy and validating games for health. Games Health J.

[27] De Freitas S, Rebolledo‐Mendez G, Liarokapis F, Magoulas GD, Poulovassilis A (2010). Learning as immersive experiences: using the four-dimensional framework for designing and evaluating immersive learning experiences in a virtual world. Brit J Educ Technol.

[28] Arnab S, Brown K, Clarke S, Dunwell I, Lim T, Suttie N, Louchart S, Hendrix M, de Freitas S (2013). The development approach of a pedagogically-driven serious game to support Relationship and Sex Education (RSE) within a classroom setting. Comput Educ.

[29] Freeman RE (1984). Strategic Management: A Stakeholder Approach.

[30] Mitchell RK, Agle BR, Wood DJ (1997). Toward a theory of stakeholder identification and salience: defining the principle of who and what really counts. Acad Manage Rev.

[31] Larson EW, Gray CF (2014). Project Management: The Managerial Process.

[32] Harrison JS, St. John CH (2013). Foundations in Strategic Management.

[33] Annetta LA (2010). The “I's” have it: a framework for serious educational game design. Rev Gen Psychol.

[34] Ferdig R (2007). Learning and teaching with electronic games. J Educ Multimed Hypermedia.

[35] Marne B, Wisdom J, Huynh-Kim-Bang B, Labat JM (2012). The six facets of serious game design: a methodology enhanced by our design pattern library. Proceedings of the 7th European Conference on Technology Enhanced Learning.

[36] Rooney P (2014). A theoretical framework for serious game design. Int J Game Based Learn.

[37] Vanden AV, De Schutter B, Geurts L, Desmet S, Wauters J, Husson J, Van den Audenaeren L, Van Broeckhoven F, Annema JH, Geerts D (2011). P-III: a player-centered, iterative, interdisciplinary and integrated framework for serious game design and development. Proceedings of the Joint Conference of the Interdisciplinary Research Group of Technology, Education, Communication, and the Scientific Network on Critical and Flexible Thinking.

[38] Yusoff A, Crowder RM, Gilbert L, Wills G (2009). A conceptual framework for serious games. Proceedings of the Ninth IEEE International Conference on Advanced Learning Technologies.

[39] Gee JP, Hayes E (2009). “No quitting without saving after bad events”: gaming paradigms and learning in the sims. Int J Learn Media.

[40] Roungas B, Dalpiaz F (2015). A model-driven framework for educational game design. Proceedings of the Games and Learning Alliance.

[41] Breuer JS, Bente G (2010). Why so serious? On the relation of serious games and learning. Eludamos J Comput Game Culture.

[42] Deci EL, Ryan RM (1985). The general causality orientations scale: self-determination in personality. J Res Pers.

[43] McKenney S, Voogt J, Kirschner PA, Superfine AC, Goldman SR, Ko ML (2022). Learning by design: nourishing expertise and interventions. Teacher Learning in Changing Contexts.

[44] L Bunt - PhD Questionnaire Video. YouTube.

[45] Petri G, von Wangenheim CG (2019). A method for the evaluation of the quality of games for computing education. Proceedings of the Congresso Brasileiro de Informática na Educação.

[46] Caserman P, Hoffmann K, Müller P, Schaub M, Straßburg K, Wiemeyer J, Bruder R, Göbel S (2020). Quality criteria for serious games: serious part, game part, and balance. JMIR Serious Games.

[47] Tsekleves E, Cosmas J, Aggoun A (2014). Benefits, barriers and guideline recommendations for the implementation of serious games in education for stakeholders and policymakers. Brit J Educ Technol.

[48] Zhonggen Y (2019). A meta-analysis of use of serious games in education over a decade. Int J Comput Games Technol.

[49] QuillBot homepage. QuillBot.

[50] ChatGPT  login. ChatGPT.

